# *Aeromonas* spp. Bacteremia in Pregnant Women, Thailand–Myanmar Border, 2011

**DOI:** 10.3201/eid1809.111592

**Published:** 2012-09

**Authors:** Paul Turner, Charlotte Willemse, Kawalee Phakaudom, Thet Wai Zin, Francois Nosten, Rose McGready

**Affiliations:** Shoklo Malaria Research Unit, Mae Sot, Thailand (P. Turner, C. Willemse, K. Phakaudom, T.W. Zin, F. Nosten, R. McGready);; Mahidol-Oxford Tropical Medicine Research Unit, Bangkok, Thailand (P. Turner, F. Nosten, R. McGready);; and University of Oxford, Oxford, UK (P. Turner, F. Nosten, R. McGready)

**Keywords:** pregnant, miscarriage, bacteremia, *Aeromonas*, bacteria, Thailand, Myanmar

**To the Editor**: *Aeromonas* spp. bacteria cause a broad spectrum of human infections (*1*). Invasive infections can be associated with exposure to contaminated water and occur frequently in patients with underlying immunosuppression or trauma but only infrequently in pregnant women (*2*). During January–May 2011, *Aeromonas* spp. bacteremia was identified in 3 pregnant Myanmar women of Karen ethnicity, who sought care at migrant/refugee clinics on the Thailand–Myanmar border.

In January 2011, a 31-year-old woman in week 12 of pregnancy sought care at the Wang Pha migrant clinic. She reported a brief history of fever, headache, abdominal pain, and vaginal bleeding. She was tachycardic and tachypneic. A complete blood count indicated mild thrombocytopenia (104 × 10^3^/μL). Ampicillin, gentamicin, and metronidazole were empirically prescribed. Ultrasonography confirmed a nonviable fetus, and products of conception were surgically evacuated. Four days later, treatment was changed to oral amoxicillin and ciprofloxacin because of clinical improvement and isolation of *A. hydrophila* from blood collected at admission. The patient recovered and was discharged after 6 days.

In March 2011, a 25-year-old primigravid woman in week 12 of pregnancy sought care at the Maela refugee camp; she had a 24-hour history of fever, chills, vomiting, diarrhea, and vaginal bleeding. She had recently sought treatment from a traditional birth attendant, who reportedly had inserted 3 sticks into the cervix and performed uterine massage. At admission, the patient was febrile and in shock. Bimanual examination revealed an open cervix with malodorous discharge. Ultrasonography revealed only products of conception. The patient received intravenous fluids and was empirically treated with ampicillin, gentamicin, and metronidazole. After surgical evacuation of the products of conception, the patient’s condition continued to deteriorate. Despite referral to the local hospital, the woman died the next day with clinical features suggestive of septic shock and disseminated intravascular coagulopathy. Complete blood count results were unavailable. *A. veronii* biovar sobria was isolated from blood collected at admission.

In May 2011, a 50-year-old woman in week 12 of pregnancy was admitted to the Wang Pha Clinic with a history of vaginal bleeding. She was febrile and in shock. Vaginal examination detected an open cervix with malodorous discharge. Complete blood count indicated reference level leukocytes and mild thrombocytopenia (116 × 10^3^ cells/µL). Blood was collected for culture. The patient received intravenous fluids and was empirically treated with ampicillin, gentamicin, and metronidazole. Products of conception were removed under ultrasonographic guidance. Three days later, treatment was changed to ceftriaxone and metronidazole because of continuing fever and positive blood culture findings (gram-positive cocci and gram-negative bacilli). These organisms were subsequently identified as *Streptococcus pyogenes* and *A. veronii* biovar sobria. The patient’s condition improved rapidly, and after 5 days she was discharged with prescriptions for oral amoxicillin and ciprofloxacin.

Blood culture isolates were provisionally identified as *Aeromonas* spp. by Gram stain, oxidase test, and API 20NE and 20E kits (bioMérieux, Marcy L’Étoile, France). The Aerokey II algorithm was used to identify the isolates to the species level (Table) (*3*). Susceptibilities to antimicrobial drugs were obtained by the agar disk–diffusion method, according to Clinical and Laboratory Standards Institute guidelines (*4*).

**Table:** Characteristics of *Aeromonas* spp. isolates from blood of 3 pregnant women, Thailand–Myanmar border, January–May 2011*

Characteristic	Patient no., organism		
	1, *A. hydrophila*	2, *A. veronii* biovar sobria	3, *A. veronii* biovar sobria
Species identification results			
Growth in 1% NaCl	+	+	+
Growth in 6% NaCl	–	*–*	–
Growth on TCBS agar (colonies)	+/– (yellow)	+/– (yellow)	+/– (green)
Glucose fermentation	+	+	+
Oxidase	+	+	+
O129 susceptibility (10 µg/150 µg)	–/–	–/–	–/–
Esculin hydrolysis	+	–	–
Indole	+	+	+
Voges-Proskauer	+	+	+
Gas from glucose (TSI)	+	+	+
Acid from arabinose	–	–	–
Cephalothin susceptibility (30 µg)	–	+	–
Antimicrobial drug susceptibilities (zone diameter, mm)			
Ampicillin (10 µg)	NA (*6*)	NA (*6*)	NA (*6*)
Ceftazidime (30 µg)	S (30)	S (31)	S (32)
Ceftriaxone (30 µg)	S (26)	S (40)	S (31)
Meropenem (10 µg)	S (30)	S (28)	S (23)
Chloramphenicol (30 µg)	S (30)	S (31)	S (28)
Ciprofloxacin (5 µg)	S (33)	S (35)	S (26)
Co-trimoxazole (25 µg)	S (24)	S (22)	S (23)
Gentamicin (10 µg)	S (20)	S (19)	S (18)

*TCBS, thiosulfate-citrate-bile salts-sucrose; O129, disks impregnated with 2,4-diamino-6,7di-iso-propylpteridine phosphate for the presumptive identification of *Vibrio* spp. from other gram-negative rods; TSI, triple sugar iron; NA, not applicable; S, susceptible (derived according to criteria from [*4*]).

One other case of invasive *Aeromonas* spp. infection during pregnancy (*A. hydrophila* sepsis at week 24 of gestation) has been reported; the focus of infection in that case was hepatobiliary, and normal pregnancy outcome was documented (*2*). For the 3 case-patients reported here, miscarriage occurred for all. Although products of conception were not submitted for culture, septic abortion was suspected clinically. Of note, *A. veronii* biovar sobria has been associated with abortion in water buffaloes (*5*). Nonmedical abortion, including the use of sticks to terminate pregnancy, is reportedly conducted on the Thailand–Myanmar border (*6*), and its use in 1 of the case-patients reported here was confirmed. Such practices predispose women to infectious complications as a result of the use of nonsterile instruments. *Aeromonas* spp. can colonize the human genital tract (*7*). Therefore, the organisms could have gained entry on contaminated abortion sticks or, if the vagina was colonized, as a result of the trauma of the procedure. An alternative hypothesis to explain our clinical findings is that fetal loss occurred as a result of *Aeromonas* spp. sepsis originating from another exposure.

The isolates were susceptible to a variety of antimicrobial drugs (Table). Empirically prescribed antimicrobial drug protocols for treatment of sepsis during pregnancy (e.g., ampicillin, gentamicin, and metronidazole or an extended-spectrum cephalosporin and metronidazole) should be effective against *Aeromonas* spp. However, if *Aeromonas* spp. are isolated, targeted treatment with a fluoroquinolone, extended-spectrum cephalosporin, or carbapenem is more appropriate (*1*).

We speculate that contamination of sticks used to induce abortion might play a role in these infections in this setting, although obtaining a definitive history to substantiate this speculation is difficult. Also, the scarcity of diagnostic microbiology laboratories in resource-poor settings results in underidentification of the causative pathogens.
